# Supplementary material for: Identifying Lymph Nodes and Their Statuses from Pretreatment Computer Tomography Images of Patients with Head and Neck Cancer Using a Clinical-Data-Driven Deep Learning Algorithm
Source: Cancers (Basel). 2023 Dec 18;15(24):5890. doi: 10.3390/cancers15245890 (PMC10741600; doi:10.3390/cancers15245890)
Supplement: Supplementary file 1 [file cancers-15-05890-s001.zip › Supplement Table 2.pptx]

## Slide 1
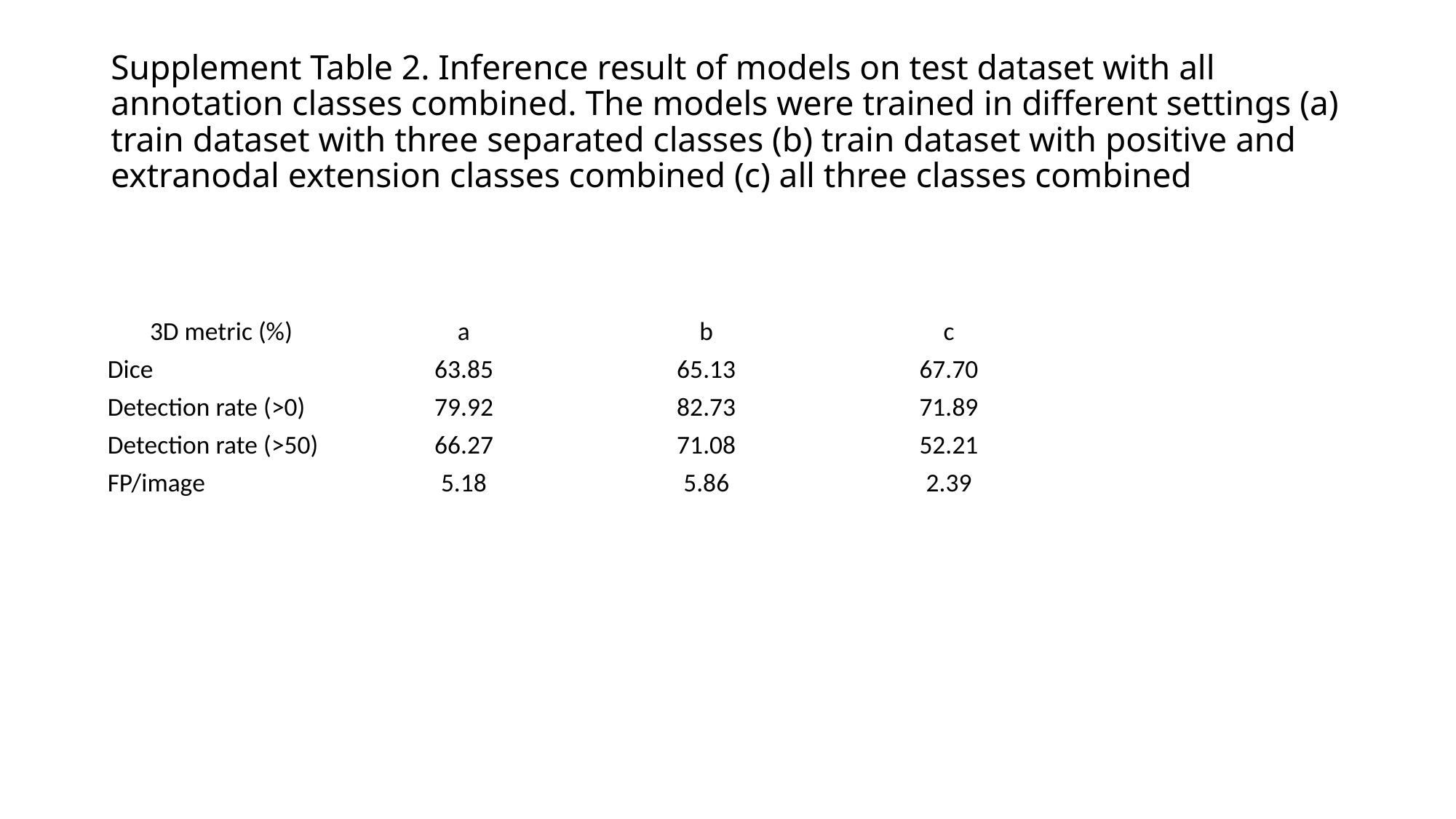

# Supplement Table 2. Inference result of models on test dataset with all annotation classes combined. The models were trained in different settings (a) train dataset with three separated classes (b) train dataset with positive and extranodal extension classes combined (c) all three classes combined
| 3D metric (%) | a | b | c |
| --- | --- | --- | --- |
| Dice | 63.85 | 65.13 | 67.70 |
| Detection rate (>0) | 79.92 | 82.73 | 71.89 |
| Detection rate (>50) | 66.27 | 71.08 | 52.21 |
| FP/image | 5.18 | 5.86 | 2.39 |
