# Supplementary material for: Identifying Lymph Nodes and Their Statuses from Pretreatment Computer Tomography Images of Patients with Head and Neck Cancer Using a Clinical-Data-Driven Deep Learning Algorithm
Source: Cancers (Basel). 2023 Dec 18;15(24):5890. doi: 10.3390/cancers15245890 (PMC10741600; doi:10.3390/cancers15245890)
Supplement: Supplementary file 1 [file cancers-15-05890-s001.zip › Supplement Table 3.pptx]

## Slide 1
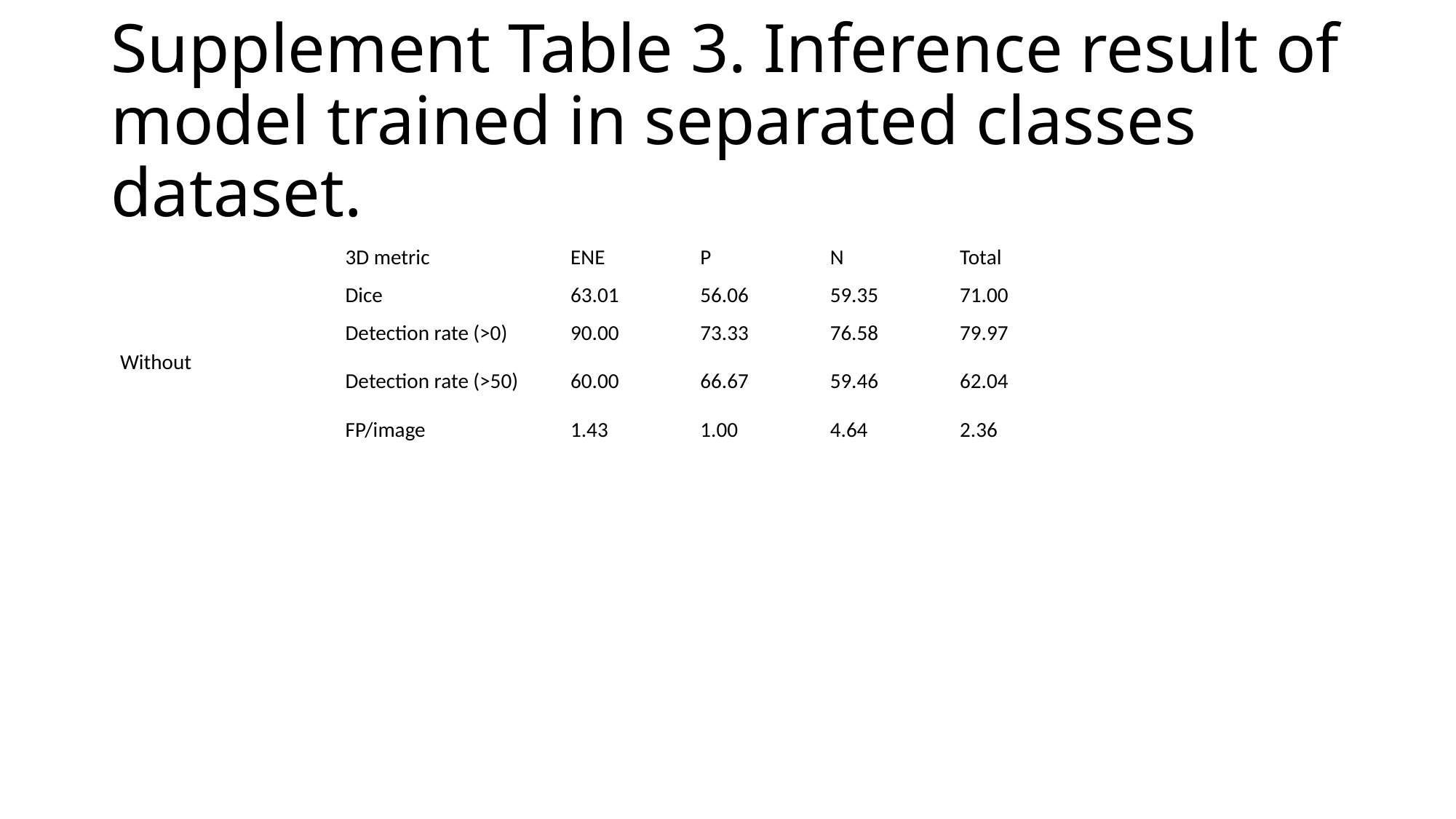

# Supplement Table 3. Inference result of model trained in separated classes dataset.
| | 3D metric | ENE | P | N | Total |
| --- | --- | --- | --- | --- | --- |
| Without | Dice | 63.01 | 56.06 | 59.35 | 71.00 |
| | Detection rate (>0) | 90.00 | 73.33 | 76.58 | 79.97 |
| | Detection rate (>50) | 60.00 | 66.67 | 59.46 | 62.04 |
| | FP/image | 1.43 | 1.00 | 4.64 | 2.36 |
