# Supplementary material for: Identifying Lymph Nodes and Their Statuses from Pretreatment Computer Tomography Images of Patients with Head and Neck Cancer Using a Clinical-Data-Driven Deep Learning Algorithm
Source: Cancers (Basel). 2023 Dec 18;15(24):5890. doi: 10.3390/cancers15245890 (PMC10741600; doi:10.3390/cancers15245890)
Supplement: Supplementary file 1 [file cancers-15-05890-s001.zip › Supplement Table 4.pptx]

## Slide 1
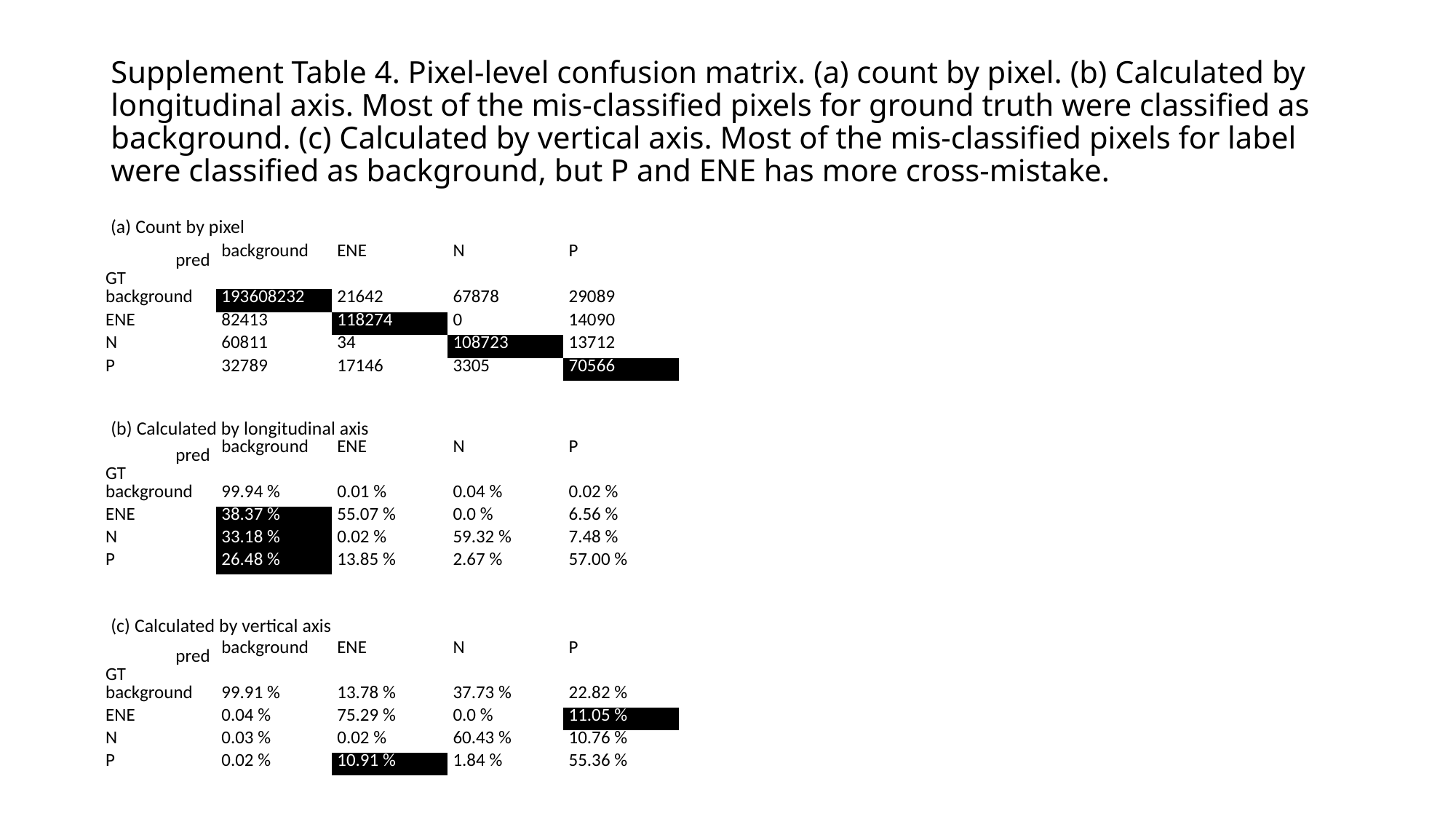

# Supplement Table 4. Pixel-level confusion matrix. (a) count by pixel. (b) Calculated by longitudinal axis. Most of the mis-classified pixels for ground truth were classified as background. (c) Calculated by vertical axis. Most of the mis-classified pixels for label were classified as background, but P and ENE has more cross-mistake.
(a) Count by pixel
| pred GT | background | ENE | N | P |
| --- | --- | --- | --- | --- |
| background | 193608232 | 21642 | 67878 | 29089 |
| ENE | 82413 | 118274 | 0 | 14090 |
| N | 60811 | 34 | 108723 | 13712 |
| P | 32789 | 17146 | 3305 | 70566 |
(b) Calculated by longitudinal axis
| pred GT | background | ENE | N | P |
| --- | --- | --- | --- | --- |
| background | 99.94 % | 0.01 % | 0.04 % | 0.02 % |
| ENE | 38.37 % | 55.07 % | 0.0 % | 6.56 % |
| N | 33.18 % | 0.02 % | 59.32 % | 7.48 % |
| P | 26.48 % | 13.85 % | 2.67 % | 57.00 % |
(c) Calculated by vertical axis
| pred GT | background | ENE | N | P |
| --- | --- | --- | --- | --- |
| background | 99.91 % | 13.78 % | 37.73 % | 22.82 % |
| ENE | 0.04 % | 75.29 % | 0.0 % | 11.05 % |
| N | 0.03 % | 0.02 % | 60.43 % | 10.76 % |
| P | 0.02 % | 10.91 % | 1.84 % | 55.36 % |
