# Supplementary figures and images for: Identifying Lymph Nodes and Their Statuses from Pretreatment Computer Tomography Images of Patients with Head and Neck Cancer Using a Clinical-Data-Driven Deep Learning Algorithm
Source: Cancers (Basel). 2023 Dec 18;15(24):5890. doi: 10.3390/cancers15245890 (PMC10741600; doi:10.3390/cancers15245890)

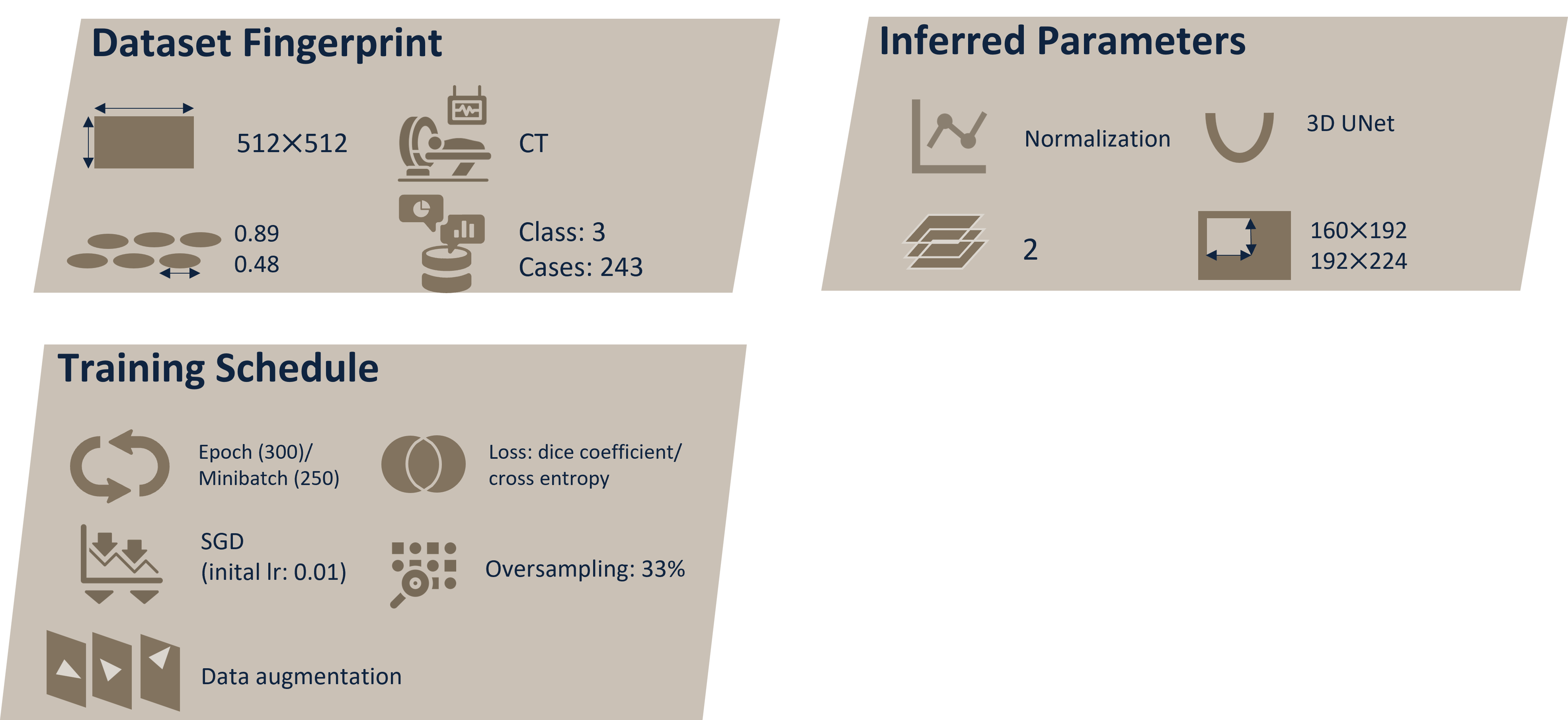

Supplement: Supplementary file 1 [file cancers-15-05890-s001.zip › Supplement Figure 1.png]

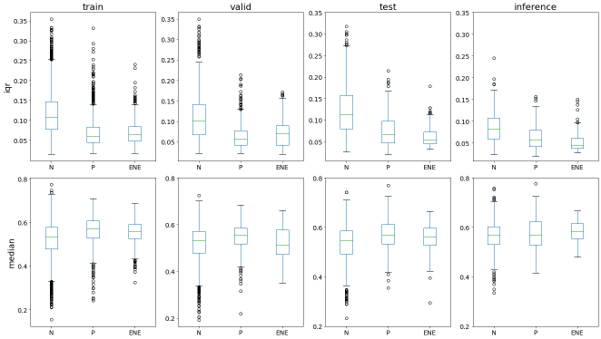

Supplement: Supplementary file 1 [file cancers-15-05890-s001.zip › Supplement Figure 2.png]

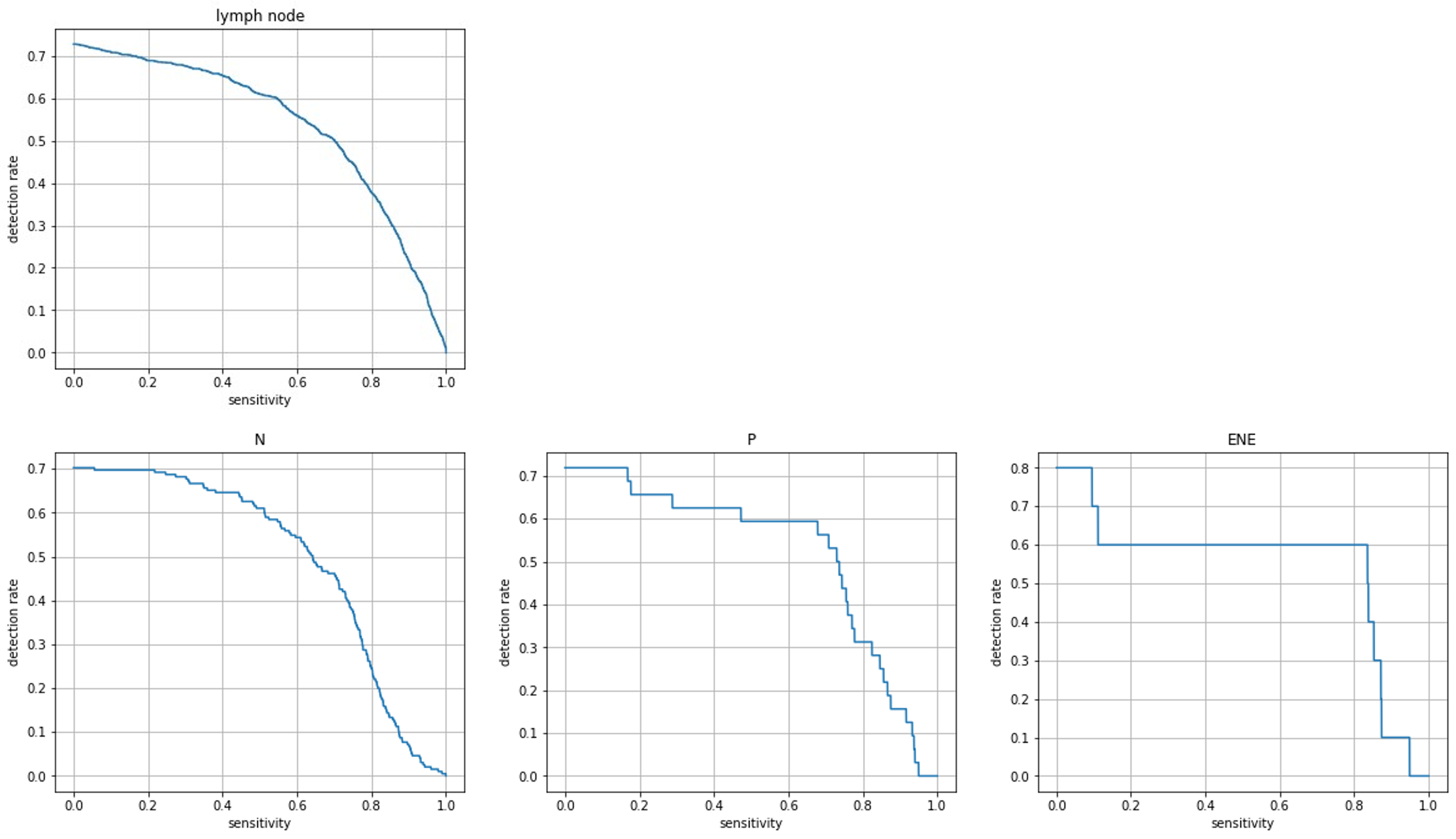

Supplement: Supplementary file 1 [file cancers-15-05890-s001.zip › Supplement Figure 3.png]

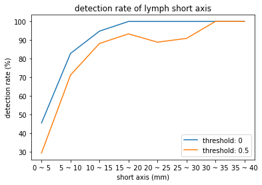

Supplement: Supplementary file 1 [file cancers-15-05890-s001.zip › Supplement Figure 4.png]
